# Understanding Factors That Predict Advance Directive Completion

**DOI:** 10.1089/pmr.2021.0073

**Published:** 2022-09-27

**Authors:** Siobhan P. Aaron, Christine Musacchio, Sara L. Douglas

**Affiliations:** ^1^ Case Western Reserve University, Frances Payne Bolton School of Nursing, Cleveland, Ohio, USA.; ^2^ Ursuline College, The Breen School of Nursing and Health Professions, Pepper Pike, Ohio, USA.

**Keywords:** advance directive, decision making, end of life, goals of care, oncology, palliative care

## Abstract

**Background:**

Advance care planning was designed for the purpose of ensuring that patients receive care at end of life (EOL) that is congruent with their wishes, goals, and values. Despite the evidence of the negative impact of not having advance directives (ADs), only one-third of adults in the United States have written ADs. Determining the patient's goals of care in the setting of metastatic cancer is vital to the delivery of high-quality healthcare. Although much is known about barriers to AD completion (e.g., the uncertainty of the disease process and trajectory, readiness of patient and family to have these discussions, and patient–provider communication barriers), little is known about the role of both patient and caregiver factors influencing AD completion.

**Objective:**

This study aimed to understand the relationship between patient and family caregiver demographic characteristics, and processes, and their influence on AD completion.

**Design:**

This study was a cross-sectional descriptive correlational design and employed secondary data analysis. The sample was composed of 235 patients with metastatic cancer and their caregivers.

**Results:**

A logistic regression analysis was performed to analyze the relationship between predictor variables and the criterion variable of AD completion. Out of the 12 predictor variables, only 2 variables (patient age and race) predicted AD completion. Of those two predictor variables, patient age made a greater and unique contribution to explaining AD completion, compared with patient race.

**Conclusion:**

There is a need for further research on cancer patients with historical low AD completion.

## Introduction

Metastatic disease progression in cancer has a significant impact upon patients and their families.^1–3^ This impact can include uncontrolled symptoms, need for advance care planning, psychosocial support, assistance with spiritual distress, need to understand prognosis and make effective treatment decisions, and the need for there to be patient and family support to prepare for the end-of-life (EOL) transition.^4–6^ It has been shown that up to 67% of patients with metastatic cancer have no documented EOL care preference, something that is typically found in an advance directive (AD) document.^7^ ADs inform patient family and health care teams regarding how patients wish to receive care toward the end of their lives.^8^ It is the documentation of a patient's goals, wishes, and values.

There has been substantial evidence regarding the benefits of completing an AD. Research has shown that ∼80% of Americans agree with the usefulness of an AD, but only 37% actually complete an AD.^9^ The use of ADs and discussions about EOL goals of care are associated with better quality of life, reduced use of nonbeneficial medical care near death, enhanced goal-consistent care, more positive family outcomes, and reduced health care costs.^10,11^ When a patient with cancer does not have an AD, it can create a myriad of problems for the family and health care team—but more importantly, for the patient. Not having an AD at EOL has been shown to prolong a patient's suffering, decrease their quality and length of life, and incur higher health care costs.^12,13^

Although the benefits of ADs are widely known, it is important to note their limitations. Various individuals prefer circumstances of care to predict their treatment preferences. Others may want their family members to make EOL decisions with insight from the circumstances when the time comes for a decision to be made.^14^ Alternatively, some people may desire to have their personal, religious, or spiritual beliefs to guide their care at EOL.^15^ Others may not understand fully the context of treatment preferences and, therefore, decide not to make a decision about EOL preferences.^16^ ADs should act as a guide to direct EOL treatment, and several conversations should be had to gain a complete context of how an individual would prefer treatment in the event they were unable to make decisions about their care.^17–19^

An AD is separate from goals-of-care discussions, as it is a legal document that serves as a guide for the surrogate health care decision maker and health care team to carry out the goals of care for a patient in the event they are unable to make their own decisions. This legal document is a form of protection that an individual possesses in which they can expect their wishes to be upheld. Furthermore, this documentation decreases stress and uncertainty in surrogate decision making regarding the election of treatment at EOL.

Despite the evidence of positive outcomes associated with ADs, the rate of AD completion has remained virtually unchanged over the past 30 years, with only 35% of patients with metastatic cancer completing ADs.^9,20^ Although much research has focused on the *outcomes* of patients with cancer who do not have ADs, few research studies have examined *factors* related to the reasons patients do not complete these documents. The small number of studies that have examined patient factors associated with not completing ADs have found that patients' lack of information, poor health care literacy, and misunderstanding about prognosis contribute to low AD completion.^21–23^

Although some studies have examined *caregiver factors* that contribute to lack of ADs, no study to date has incorporated *both* patient and caregiver factors in the same study. Therefore, the purpose of this study was to describe the relationship between patient and family caregiver demographic characteristics and processes (care preferences and goals) that influence presence or absence of AD completion (a reflection of EOL decision making).

## Sample and Methods

Prior to data analysis, this study was granted a waiver of informed consent by the University Hospitals of Cleveland IRB due to study procedures of a retrospective review of de-identified data. This study design was cross-sectional, descriptive, and correlational, and employed secondary data analysis. Data that were used for these analyses came from a larger parent study conducted from 2014 to 2018. The parent study (Mapping Complex Influences on Aggressiveness of End-of-Life Cancer Care NINR: NRO14856) obtained self-reported data from patients with metastatic solid tumor cancer, their caregivers, and their oncologists, and focused on the examination of the relationships between factors from these groups upon the receipt of aggressive EOL care.^24^ For the purposes of this substudy, patient and caregiver characteristic variables, their EOL values and goals, and the patient's decision regarding an AD were used. The outcome of interest was the presence or absence of AD completion.

This study used self-report measures from *patients and caregivers only*. Goals of care were measured using a single visual analog scale wherein scores ranged from 0 (“quality of life is all that matters”) to 100 (“length of life is all that matters”).^24^ The tool has demonstrated excellent psychometric properties.^25^ AD completion was measured using a chart review of the electronic medical record (EMR). Patients who had documentation in their EMR of either a living will, durable power of attorney for health, or both, were considered to have completed an AD. For the purposes of this study, baseline data were used for all analyses.

Eligibility criteria were (1) patient had a diagnosis of stage IV solid tumor of lung or gastrointestinal (GI) cancer, (2) patient identified an informal (unpaid) caregiver who provided care and/or support, and (3) both patient and caregiver could speak English. Logistic regression analysis was used to examine the relationship between patient and caregiver variables and AD completion (yes/no). Predictor variables were patient age, race, income, education level, religiosity, and cancer type and symptom burden; caregiver age, income, religiosity, and physical health; and patient goals of care and caregiver goals of care for the patient.

## Results

There were 235 patient–caregiver dyads enrolled in the study. Patients were, on average, 65 years of age (standard deviation [SD] = 11). Almost half of the patients had GI cancer. In general, patients were White, of Catholic religion, and married. A little over half of the patients were retired, and almost half reported an average household income category of $50,000 or greater. The average age of caregivers in the sample was 59 years of age (SD = 12). Most caregivers were female, White, and married to the patient. Approximately half of all caregivers were employed, and over half reported an average household income category of $50,000 or greater.

Key demographic and clinical characteristics of patients and caregivers are given in Table 1. Out of the patient sample, 61% had documentation of an AD. Of the 61% of the patient sample who obtained an AD, 90% were White and 70% were in the highest income category.

**Table 1.** Patient and Caregiver Demographics

	Patient	Caregiver
Characteristic	*N* (%)	*N* (%)
Age (years)	235 (100)	235 (100)
21–39	2 (0.9)	18 (7.7)
40–49	18 (7.6)	28 (11.9)
50–59	48 (20.4)	63 (26.8)
60–69	90 (38.3)	81 (34.5)
70–79	52 (24.7)	34 (14.4)
80–88	19 (8.1)	11 (4.7)
Race/ethnicity		
White	195 (83.0)	197 (83.8)
African American	34 (14.5)	32 (13.6)
Asian	2 (0.9)	2 (0.9)
Hispanic	4 (1.7)	1 (0.4)
Racial groups		3 (1.3)
White	195 (82.9)	
Non-White	40 (17.1)	197(83.8)
Gender		38 (16.2)
Female	107 (45.1)	
Male	130 (54.9)	163 (69.4)
Cancer type		72 (30.6)
GI	103 (43.8)	
Lung	82 (34.9)	177 (75.3)
Pancreatic	50 (21.3)	58 (24.7)
Religion		
Catholic	84 (35.7)	88 (37.6)
Protestant	73 (31.1)	62 (26.5)
Jewish	12 (5.1)	14 (6.0)
No preference	32 (13.6)	32 (13.7)
Other	33 (14.0)	38 (16.2)
Marital status		
Married	172 (73.2)	186 (79.1)
Not married	63 (26.8)	49 (20.9)
Employment status		
Employed	54 (23.1)	151 (64.3)
Retired	120 (51.3)	39 (16.6)
Disabled	33 (14.1)	17 (7.2)
Not employed	20 (8.5)	8 (3.4)
Other	7 (3)	20 (8.5)
Annual household income		
$20,000 or less	37 (15.7)	117 (49.8)
$21,000 to $49,999	66 (28.1)	91 (38.7)
$50,000 or greater	110 (46.8)	7 (3)
Relationship with patient		
Partner/spouse		151 (64.3)
Child		39 (16.6)
Sibling		17 (7.2)
Parent		8 (3.4)
Other		20 (8.5)

GI, gastrointestinal.

A logistic regression analysis was performed to analyze the relationship between predictor variables and the criterion variable of AD completion (yes/no). Out of the 12 predictor variables, only 2 variables (patient age and race) predicted AD completion; see Table 2 for logistic regression results. Of those two predictor variables, patient age made a greater and unique contribution to explaining AD completion, compared with patient race.

**Table 2.** Logistic Regression Forecasting Advance Directive Documentation Based on Patient and Caregiver Characteristics and Processes

Variable	***B***	SE	Wald	***p***	OR	95% CI OR
PT age	0.08	0.02	13.91	0.001^***^	1.09	1.041–1.13
PT annual income	0.15	0.29	0.29	0.59	1.17	0.663–2.05
PT religiosity scale	0.04	0.05	0.63	0.43	1.04	0.95–1.13
Edmonton Symptom Burden Scale	−0.01	0.01	1.67	0.20	0.99	0.97–1.01
Cancer type	−0.28	0.23	1.45	0.23	0.76	0.48–1.19
CG age	0.00	0.02	0.01	0.93	1.00	0.97–1.04
CG annual income	0.02	0.34	0.001	0.96	1.02	0.52–1.98
PROMIS CG physical health scale	−0.01	0.01	0.55	0.46	0.99	0.98–1.01
CG religiosity scale	−0.07	0.05	1.88	0.17	0.93	0.84–1.03
Patient focus of care scale	0.01	0.01	1.70	0.19	1.01	1.00–1.02
Caregiver focus of care scale	−0.01	0.01	0.78	0.38	0.99	0.98–1.01
PT race	−1.0*2*	0.*50*	4.1*5*	0.04^*^	0.36	0.14–0.97

Patient race is coded as 0 for non-Caucasian and 1 for Caucasian. AD completion is coded as 0 for yes and 1 for no. 95% CI is confidence interval around odds ratio upper and lower.

^*^ *p* < 0.05. ^***^p < 0.001.

ADs, advance directives; CG, caregiver; CI, confidence interval; OR, odds ratio; PT, patient.

For every year that the patient's age increased, the likelihood of AD completion was increased. As the patient's age increased, the patient was 1.1 times more likely to have a completed AD (odds ratio [OR] = 1.088, confidence interval [95% CI 1.041–1.137], *p* = 0.0001). In addition, non-White patients were less likely to have a completed AD compared with White patients (OR = 0.362, 95% CI [0.136–0.963], *p* = 0.04).^7,23,25–27^

## Discussion

Our study demonstrated that patient age and race predicted AD completion—a finding that confirms the work of other studies.^7,25–27^ The percentage of White and older individuals who completed ADs found in our study supported the work of prior research as well. Unlike prior research,^25,28–32^ we did not find any other factors (e.g., religiosity, goals of care, income, and education level) that predicted AD completion, nor did we find any caregiver factors that significantly forecasted AD completion.

The percentage of individuals in this sample who had completed ADs was higher than the general population that tends to have completion rates of ∼32%.^7^ Owing to the sample being one with metastatic cancer, this is consistent with the literature in that several studies have shown that patients with chronic or terminal disease having a higher rate of AD completion than those who do not.^31^ Notably, this study had a higher AD completion rate (61%) than similar studies consisting of oncology patients (30–40%).^33–39^ In addition, 54% of this sample was over the age of 65 years old, and mostly White (84%), which also plays a factor into higher rates of AD completion.

### Limitations

There are four study limitations. First, we used convenience sampling, thus limiting generalizability of study findings. Second, our sample was composed solely of patients with stage IV solid tumor cancer, and our study can only be generalized to this population.^26,38^ Our sample was also composed of patients who were older than those reported in prior studies.^34,40,41^ This could have influenced our AD completion rate because age has been shown to predict completion, and our sample showed a higher AD completion rate than the national average.

The third limitation was that this was a cross-sectional design, and causation cannot be inferred. Finally, the dependent variable of AD completion was determined solely by documentation in the patient's EMR and may not be an accurate measure of the phenomenon. Future research should include interventions for new approaches to understand the dynamics of how patients make the decision to complete an AD or not.

## Conclusion

Our study analyzed the relationships between patient and caregiver factors and patient AD completion. The results demonstrate that AD completion rates have not changed much over the past 10 years, and there remains a need for ongoing work to understand factors and barriers to AD completion. The study showed AD completion rates a little higher than the national average, but consistent with other studies that were composed of cancer populations. Moreover, patients in the study who completed ADs, shown similar completion AD completion rates to those in other studies. This highlights the need to study cancer patients in populations who traditionally have lower rates of AD completion.

## Abbreviations Used

ADs: advance directives
CI: confidence interval
EMR: electronic medical record
EOL: end-of-life
GI: gastrointestinal
OR: odds ratio
SD: standard deviation
